# VIRTUAL REALITY FOR TREATMENT OF DEPRESSION IN AN OLDER ADULT WITH PARKINSON’S DISEASE

**DOI:** 10.1093/geroni/igae098.2645

**Published:** 2024-12-31

**Authors:** Sabine Lohmar, Amy Fiske

**Affiliations:** West Virginia University, Morgantown, West Virginia, United States; West Virginia University, Morgantown, West Virginia, United States

## Abstract

Research on the effectiveness of virtual reality, an interactive computer-generated environment, as a form of behavioral activation for older adults with depression, has been minimal but promising (Yen & Chui, 2021). A case study of an older male nursing home resident with Parkinson’s Disease who uses a wheelchair is presented to provide clinical evidence for the value of using virtual reality in treating depression in older adults with physical limitations. Virtual reality sessions consisting of immersive and interactive outdoor experiences (e.g., fishing, hunting, hiking, kayaking) were conducted for an average of 20 minutes once every two weeks for six months. The Geriatric Depression Scale Shortened Form (GDS-S) was administered before, during, and after the use of virtual reality to assess depressive symptoms. The patient’s depressive symptoms improved from intake (GDS-S = 11) to termination (GDS-S = 4). Moreover, subjective reports from the patient indicated an overall elevation in mood, perceived enjoyment, increased motivation, and reduced pain. This case study demonstrates the potential benefits of virtual reality for physically limited older adults with depression who may face barriers, for example, due to a movement disorder, to engaging in previously enjoyed activities.

